# Health access and prevention under Arkansas' market-based Medicaid expansion

**DOI:** 10.1093/haschl/qxag045

**Published:** 2026-02-25

**Authors:** Robert J Skinner, Christopher J Louis, Colleen Florio, Jessica Lang, Kevin N Griffith

**Affiliations:** Department of Health Policy, Vanderbilt University Medical Center, Nashville, TN, United States; Department of Health Law, Policy & Management, Boston University School of Public Health, Boston, MA, United States; Public Consulting Group, Boston, MA, United States; Lang Health Insights, LLC, Brookline, MA, United States; Department of Health Policy, Vanderbilt University Medical Center, Nashville, TN, United States; Partnered Evidence-Based Policy Resource Center, VA Boston Healthcare System, Boston, MA, United States

**Keywords:** access to care, Medicaid, insurance, health reform

## Abstract

**Introduction:**

Arkansas expanded Medicaid in 2014 using a Section 1115 waiver that included premium assistance for beneficiaries to purchase private plans through the state's marketplace. The waiver was modified in 2017 to require partial premium payments and implemented work requirements in mid-2018, which were later rescinded by federal court order. We compared healthcare access and preventive service utilization in Arkansas vs states with traditional Medicaid expansions.

**Methods:**

Using Behavioral Risk Factor Surveillance System data from 2011 to 2021, we applied difference-in-differences analysis to examine low-income non-elderly adults aged 18-64 in Arkansas and 3 demographically similar traditionally expanding states (Kentucky, Ohio, West Virginia). Outcomes included insurance coverage, usual source of care, annual checkups, cost-related care avoidance, and flu vaccination.

**Results:**

Compared to traditional expansion states, Arkansas' expansion was associated with greater increases in insurance coverage (+3.5% points, *P* < 0.001) and annual checkups (+6.1pp, *P* < 0.001). Both groups showed similar improvements in usual source of care and flu vaccination rates, and comparable reductions in avoided care due to cost.

**Conclusion:**

Pre-existing gaps between Arkansas and traditional expansion states narrowed for insurance coverage and rates of annual checkups, while other outcomes improved in Arkansas at levels comparable to those observed in traditional expansion states.

## Introduction

Arkansas implemented a unique Section 1115 waiver to expand Medicaid through the Health Care Independence Program (HCIP) on January 1st, 2014. HCIP increased income eligibility for Arkansas residents earning up to 138% of federal poverty level (FPL).^1^ Newly eligible Arkansans were given premiums subsidies, paid directly by the state to insurers, to purchase insurance through private qualified health plans (QHPs) on the Healthcare.gov Marketplace.^2,3^ In contrast, traditional Medicaid expansion states liberalized access to their existing Medicaid fee-for-service or managed care offerings. Arkansas' market-driven approach allowed enrollees to maintain Marketplace coverage after leaving Medicaid, potentially reducing coverage disruptions.^4^

Starting in 2017, Arkansas received approval for a waiver extension and modification which they now call Arkansas Works (ARWORKS). Several features of ARWORKS may negatively impact insurance coverage and access compared to traditional state Medicaid expansions. Arkansas became the first state to implement work requirements, albeit briefly, for Medicaid beneficiaries aged 30-49 in June 2018 and disenrolled approximately 18 000 adults before these requirements were suspended by a federal judge in April 2019.^5^ The ARWORKS program also required enrollees who make more than 100% FPL to make monthly premium payments equal to 2% of household income.^6^ Prior research from other states demonstrates that cost-sharing and premium requirements may result in reduced uptake of health insurance and subsequent receipt of healthcare.^7,8^

Recent federal policy actions underscore the importance of evaluating Arkansas' waiver. In 2025, Congress enacted the One Big Beautiful Bill Act (H.R. 1), which for the first time established national Medicaid work requirements and eliminated the role of Section 1115 waivers as the mechanism for implementing such requirements at the state level. At the same time, congressional debates include broader reforms to Medicaid financing and increased autonomy for states. Against this backdrop, understanding the effects of Arkansas' market-based waiver is critical for informing discussions about which Medicaid design features may meaningfully expand access to care, and which may risk undermining it.

Additionally, Arkansas' novel 1115 waiver may represent an attractive alternative for the 10 states that have not adopted Medicaid expansion.^9^ Early studies of Arkansas' unique expansion reported improvements in coverage, access, self-reported health,^10^ postpartum care, and depression treatment.^11,12^ Stimpson et al. (2020) found reduced uninsurance rates during the first 3 years,^13^ while others documented the deleterious effects of work requirements.^5,14,15^ Further, Medicaid beneficiaries assigned to QHPs had higher preventive service utilization,^16^ greater patient satisfaction,^17^ and broader access to high-quality providers compared to those in traditional Medicaid.^18,19^ The large increase in individual plan enrollment following Arkansas's unique Medicaid expansion was associated with lower negotiated hospital inpatient prices for those plans, consistent with insurers' increased bargaining leverage in the market.^20^

While prior evaluations advanced understanding, most focused on short-term impacts or narrow outcomes. Few studies benchmarked Arkansas against multiple traditional expansion states over an extended post-expansion period. In this paper, we identify changes in self-reported access to care and preventive service utilization associated with Arkansas' waiver implementation. We also identify how changes in these outcomes among Arkansas' residents compare to residents of states which experienced traditional Medicaid expansions.

## Data and study population

This cross-sectional study was considered exempt by Vanderbilt University Medical Center's institutional review board and adheres to the Strengthening the Reporting of Observational Studies in Epidemiology (STROBE) reporting guideline for cross-sectional studies. Institutional policy provides a waiver of informed consent because the research, which used publicly available deidentified data, could not be practicably carried out otherwise. We used data from the 2011-2021 Behavioral Risk Factor Surveillance System (BRFSS), a large random-digit-dialing telephone survey that collects data on health-related behaviors, conditions, and use of care.^21^ The BRFSS is conducted nationwide by the Centers for Disease Control (CDC) in partnership with all 50 states, the District of Columbia, and 3 US territories.

Our sample included non-elderly community dwelling adults ages 18 through 64, excluding the Medicare-eligible population who are not a target of these healthcare reforms. We converted household income categories to their midpoints, then to FPL percentage based on household size and federal guidelines, following an approach from previous literature.^4,22^ We restricted our sample to individuals who earned equal to or under 138% of the FPL each year. Thus, our calculated FPL measure is a proxy for Medicaid eligibility.

We further limited our study population to individuals in Arkansas and the 3 traditional expansion (TE) states that were most demographically similar to Arkansas according to the State Similarity Index: Kentucky, Ohio, and West Virginia.^23^ There is a vast literature documenting the effects of traditional expansion on access and preventive service utilization.^24,25,26^ Thus, our choice to compare Arkansas to traditional Medicaid expansion states is conceptually similar to a noninferiority trial, a common approach in clinical research used to determine whether a new intervention is not substantially worse than an established standard.^27^

### Study variables

We assessed the associations between Arkansas' Medicaid waiver and 5 self-reported measures of access to care and preventive service utilization (Table 1). Our outcomes were insurance coverage, having a personal doctor, avoiding care due to cost, receipt of a routine checkup, and flu vaccination. These measures are commonly used in Medicaid studies^28-32^ and show high reliability and validity.^33^

**Table 1.** List of study outcomes.

Outcome	Survey question	Response format	2011-2013 baseline rate (%)		SMD
			Traditional expansion states	Arkansas	
Has Insurance Coverage^a^	“Do you have any kind of health care coverage, including health insurance, prepaid plans such as HMOs, or government plans such as Medicare, or Indian Health Service?”	Yes/No	66.3	51.1	0.314
Has a Personal Doctor	“Do you have one person (or a group of doctors) that you think of as your personal health care provider?”	Yes/No	69.4	64.8	0.098
Avoided Care Due to Cost	“Was there a time in the past 12 months when you needed to see a doctor but could not because you could not afford it?”	Yes/No	33.5	42.8	0.193
Last Routine Checkup^b^	“About how long has it been since you last visited a doctor for a routine checkup? [A routine checkup is a general physical exam, not an exam for a specific injury, illness, or condition.]”	Within past year, within past 2 years, within past 5 years, 5 or more years ago	62.1	50.8	0.230
Flu Vaccine	“During the past 12 months, have you had either flu vaccine that was sprayed in your nose or flu shot injected into your arm?”	Yes/No	28.4	26.6	0.041

**Source:** Authors' analysis of data from low-income adult respondents to the 2011-2021 Behavioral Risk Factor Surveillance System (BRFSS).

^a^Starting in 2021, this question was modified to “What is the current primary source of your health insurance?” Respondents could identify source of insurance (eg, Medicaid, Medicare, employer) or state they were uninsured. Responses were binary coded to insured/uninsured for consistency with earlier years.

^b^This question was binary coded to indicate whether the respondent received a routine checkup within the past year.

SMD = standardized mean difference.

Study covariates included a variety of demographic and socioeconomic characteristics that have previously been associated with health care access and service utilization including respondent age (grouped into 5-year categories), marital status (married vs unmarried), education (college graduate, some college, no college), sex (male vs female), employment status (employed, unemployed, not in the labor force), household size (count of children and adults in household), veteran status (yes vs no), and homeownership status (own vs rent). Our analyses also include fixed effects for state of residence, survey month, and whether the survey was conducted via landline or cell phone.^34^ A full list of covariates and their question texts are available in Supplementary Appendix A1. Covariate-level missingness ranged from 0% to 1.2%; we used multiple imputation using additive regression, bootstrapping, and predictive mean matching to replace missing covariate values and reduce the potential for non-response bias.^21,35^ Outcome-level missingness ranged from 0.3% to 7.3% (flu vaccination); following best practices for non-linear regression models, outcomes were not imputed.^36^

## Analytic approach

Our analysis proceeded in 6 steps. We first calculated descriptive statistics for covariates and outcomes in both Arkansas and TE states and differences were assessed using standardized mean differences (SMDs). Second, we described annual trends in our self-reported measures of access to care and preventive service utilization. Third, we used covariate-adjusted regression models to assess changes over time in outcomes, estimating separate (stratified) models for Arkansas and TE states. Our models included a “baseline” period (2011-2013), an initial waiver period (2014-2016), and a waiver extension period (2017-2021).

Fourth, we estimated adjusted difference-in-difference (DID) models to compare changes in outcomes between Arkansas and TE states. We used data from the baseline period to test the parallel trends assumption, testing for differences in linear time trends between Arkansas and TE states after covariate adjustment. The DID estimates represent the difference in pre-to-post changes in outcomes between Arkansas and comparison states, rather than changes within Arkansas alone. As a result, statistically insignificant DID estimates may occur if the observed changes in Arkansas did not differ from those observed in other states, even when absolute levels changed for both groups. We also estimated event study models to compare outcomes during the pre-expansion period. If the parallel trends assumption is met, our DID approach ensures all group-invariant time trends or time-invariant covariate differences between groups do not affect our results.^37^

Fifth, as a supplemental analysis, we replaced our comparison group with the 12 states that did not expand Medicaid by December 31st, 2021: Alabama, Florida, Georgia, Kansas, Mississippi, North Carolina, South Carolina, South Dakota, Tennessee, Texas, Wisconsin, and Wyoming.

Lastly, we conducted several sensitivity analyses to check the robustness of our results. These included:

1. Removal of 2020-2021 data due to potential confounding effects of the COVID-19 pandemic.
2. Inverse probability of treatment weighting (IPTW) to increase the demographic similarity of Arkansas and TE states. The propensity model included our full set of study covariates, and the outcome was a binary indicator taking on a value of one if the respondent resided in Arkansas, zero otherwise.
3. Comparison to 15 traditional expansion (TE) states that underwent non-waivered Medicaid expansions on January 1st, 2014: California, Colorado, Connecticut, Illinois, Kentucky, Minnesota, Nevada, New Jersey, New Mexico, North Dakota, Ohio, Oregon, Rhode Island, Washington, and West Virginia. Several states provided health coverage to households with incomes at or above 100% of poverty before the ACA's Medicaid expansion and were excluded from this analysis.^38^
4. Re-analysis without multiple imputation of missing covariates, using only complete survey responses.

All models were estimated using logistic regressions with BRFSS sampling weights and standard errors clustered at the state level. We converted the resulting odds ratios and report results as average marginal effects to aid interpretability; all regression estimates should be interpreted as percentage point changes in the likelihood of experiencing the outcome. All analyses were conducted using R Statistical Software 4.4.2 (R Foundation for Statistical Computing, Vienna, Austria). Regression specifications and additional details are provided in the Supplementary Appendix. Analyses were conducted from July 2023 through January 2026.

## Study results

Our sample included 62 425 unweighted respondents across Arkansas (*N* = 9890) and our 3 similar TE states (*N* = 52 535) from 2011 to 2021 with household incomes under 138% FPL (Table 2). For brevity, when we mention “Arkansans” we are specifically referencing this low-income group.

**Table 2.** Characteristics of the study sample (N = 62 425).

Variable	Traditional expansion states,^a^ No. (weighted %)	Arkansas, no. (weighted %)	SMD
Sex			
Female	32 275 (55.5)	6227 (54.5)	0.019
Male	20 260 (44.5)	3663 (45.5)	
Marital status			
Married	18 195 (32.5)	3529 (36.7)	0.089
Unmarried	34 340 (67.5)	6361 (63.3)	
Household income			0.113
Less than $10 000	10 705 (19.5)	1983 (19.0)	
$10 000 to $15 000	9409 (15.1)	1859 (17.0)	
$15 000 to $20 000	10 692 (19.1)	2188 (21.5)	
$20 000 to $25 000	10 182 (20.5)	1957 (20.1)	
$25 000 to $35 000	6855 (14.9)	1200 (14.1)	
$35 000 +	4692 (11.0)	703 (8.3)	
Race			
White	43 318 (74.9)	6215 (62.5)	0.317
Black	5197 (16.0)	2367 (21.2)	
Hispanic	1264 (4.5)	649 (11.4)	
Other	1326 (2.9)	342 (3.0)	
Multiracial	1430 (1.7)	317 (1.8)	
Age			
18 to 24	4521 (17.2)	754 (17.9)	0.089
25 to 34	8451 (24.0)	1535 (24.8)	
35 to 44	9563 (19.9)	1815 (21.7)	
45 to 54	12 546 (19.0)	2367 (18.9)	
55 to 64	17 454 (19.9)	3419 (16.7)	
Education group			
College grad	6484 (8.1)	8686 (7.1)	0.039
Not a college grad	46 051 (91.9)	1204 (92.9)	
Employment group			
Unemployed	6107 (13.3)	1107 (13.5)	0.027
Employed	20 248 (45.6)	3887 (46.7)	
Not in labor force	26 180 (41.1)	4896 (39.8)	
Survey modality			
Landline	26 692 (36.9)	5277 (26.7)	0.221
Mobile phone	25 573 (63.1)	4613 (73.3)	
Veteran status			
Veteran	3773 (6.7)	762 (7.2)	0.188
Non-Veteran	48 762 (93.3)	9128 (92.8)	

**Source:** Authors' analysis of data from low-income adult respondents to the 2011-2021 Behavioral Risk Factor Surveillance System (BRFSS). Percentages incorporate BRFSS post-stratification weights and may not sum to 100 due to rounding.

SMD = Standardized mean difference.

^a^Traditional expansion states included Kentucky, Ohio, and West Virginia.

The average household size included 3.1 adults (SD 2.1) and 1.1 minor children (SD 1.6). Unweighted, a majority of our sample was female (61.7%), unmarried (65.2%), White (79.3%), and non-college graduates (87.7%). After weighting, Arkansans differed from TE state residents in their distribution of household incomes (SMD 0.113), race/ethnicity (SMD 0.317), survey modality (SMD 0.221), and veteran status (SMD 0.188). Prior to the ACA, Arkansans were significantly less likely to have health insurance coverage, have a usual source of care, receive annual checkups, and had higher rates of avoided care due to cost compared residents of similar TE states.

There were no statistically significant differences in linear time pre-trends between Arkansas and TE states for any study outcomes (*P*-values ranged from 0.5761 to 0.9833). Event study graphs are provided in Supplementary Appendices A2-A6 and showed only minor differences.

In subsequent sections, we distinguish between within-group changes over time and between-group differences in those changes. Changes reported for Arkansas or traditional expansion states describe absolute percentage-point differences from baseline within each group. By contrast, DID estimates quantify whether the magnitude of change in Arkansas differed from the change observed in comparison states over the same period.

### Insurance coverage

**Figure 1.**
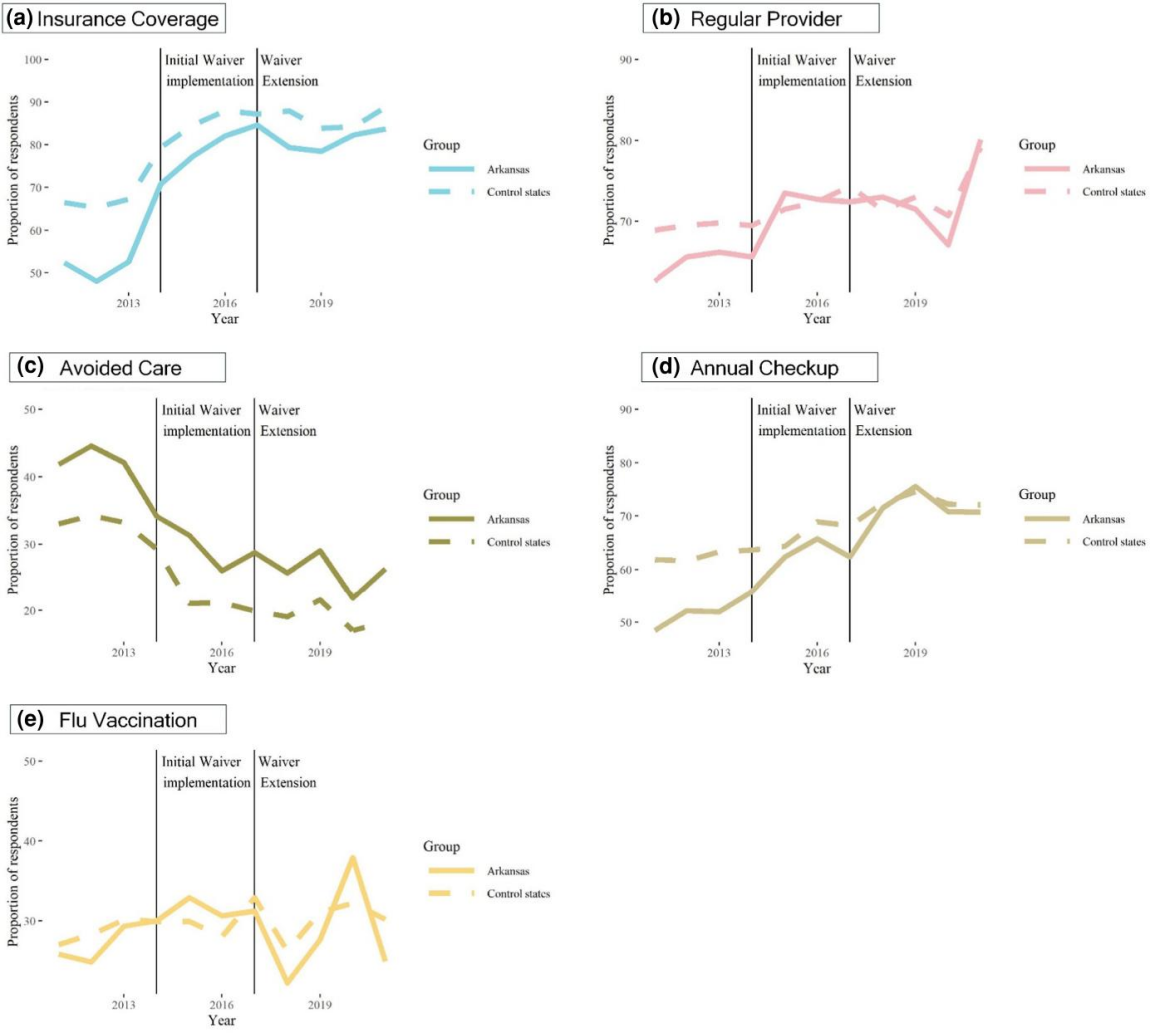
Trends in health care access for Arkansas and 3 traditional expansion states, 2011-2021. Source: Authors' analysis of data from low-income adult respondents to the 2011-2021 Behavioral Risk Factor Surveillance System (BRFSS). Notes: The figure displays weighted unadjusted rates of study outcomes. Traditional expansion states included Kentucky, Ohio, and West Virginia.

Prior to expansion, Arkansans reported lower rates of health insurance coverage compared to TE states (Figure 1 and Supplementary Appendix A7). These differences decreased throughout the study period, except in 2018 when Arkansas briefly imposed work requirements. For example, Arkansans were 14.1% points (pp) less likely to report having health coverage in 2011 compared to residents of TE states. This difference decreased to 5.1pp by 2021. Arkansas' insurance rates increased by 20.2pp (95% CI 17.3 to 23.2) during the initial waiver period (2014-2016) and an additional 6.2pp (95% CI 3.2 to 9.3) during the waiver extension period (2017-2021) (Table 3). In our DID model, Arkansas' expansion was not associated with differential changes in insurance coverage during 2014-2016. However, we observed significant increases from the baseline period during 2017-2021 (+4.7pp, 95% CI 1.5 to 7.8) and over the full post-expansion period (+3.5pp, 95% CI 0.7 to 6.2).

**Table 3.** Adjusted changes in health care access for Arkansas and 3 traditional expansion states, 2011-2021.

		Estimated differences					
		Initial waiver vs baseline		Waiver extension vs baseline		Waiver extension vs initial waiver	
Outcome	Arkansas/TE^a^	Estimate (95% CI)^b^	Difference (95% CI)^c^	Beta^b^	Difference^c^	Beta^b^	Difference^c^
Have Insurance Coverage	Arkansas	20.1*** (17.2, 23.0)	2.2 (−0.9, 5.4)	26.3*** (23.4, 29.1)	4.7** (1.5, 7.8)	6.1*** (3.1, 9.2)	2.5 (−0.7, 5.7)
	TE	15.5*** (14.0, 17.0)	–	18.5*** (17.1, 19.9)	–	3.0*** (1.5, 4.5)	–
Have Personal Doctor	Arkansas	5.3** (1.8, 8.8)	1.2 (−2.8, 5.2)	7.9*** (4.5, 11.3)	1.5 (−2.4, 5.3)	2.6 (−0.5, 5.7)	0.3 (−3.2, 3.8)
	TE	4.2*** (2.4, 5.9)	–	6.5*** (4.8, 8.2)	–	2.3** (0.8, 3.9)	–
Avoided Care Due to Cost	Arkansas	−10.3*** (−13.7, −6.9)	−1.0 (−4.5, 2.5)	−14.3*** (−17.5, −11.1)	0.8 (−2.5, 4.2)	−4.0* (−7.2, −0.8)	1.9 (−1.4, 5.2)
	TE	−8.0*** (−9.6, −6.5)	–	−13.4*** (−14.9, −11.9)	–	−5.3*** (−6.9, −3.8)	–
Last Routine Checkup	Arkansas	10.1*** (6.5, 13.6)	4.6* (0.6, 8.5)	19.3*** (16.0, 22.6)	7.3*** (3.5, 11.1)	9.3*** (5.9, 12.6)	2.8 (−0.9, 6.4)
	TE	5.2*** (3.4, 7.0)	–	11.4*** (9.7, 13.1)	–	6.2*** (4.5, 7.9)	–
Flu Vaccine	Arkansas	4.5* (1.0, 8.0)	2.7 (−1.3, 6.6)	2.4 (−1.0, 5.8)	0.0 (−3.8, 3.8)	−2.1 (−5.2, 1.1)	−2.7 (−6.2, 0.9)
	TE	1.9* (0.1, 3.6)	–	2.4** (0.7, 4.1)	–	0.6 (−1.0, 2.1)	–

**Source:** Authors' analysis of data from low-income adult respondents to the 2011-2021 Behavioral Risk Factor Surveillance System (BRFSS). The table displays regression-adjusted percentage-point changes in outcomes during each period. Regression estimates are adjusted for respondent age, marital status, education, employment status, household size, veteran status, sex, household income, homeownership status, survey month, and whether the survey was conducted via landline or cell phone. The “Baseline” period includes 2011-2013, the “Initial Waiver” period includes 2014-2016, the “Waiver Extension” period includes 2017-2021.

*P < 0.05, **P < 0.01, ***P < 0.001.

^a^Traditional expansion (TE) states included Kentucky, Ohio, and West Virginia.

^b^Results from logistic regression models stratified by outcome and AR/TE group.

^c^Difference-in-difference estimates for changes in Arkansas and TE states over time. All models incorporated BRFSS sampling weights with standard errors clustered at the state level.

### Had a personal healthcare provider

In 2011, Arkansans were 6.3pp less likely to report having a personal doctor than respondents from TE states in unadjusted comparisons. By 2021, Arkansans were 1.0pp more likely to report having a personal doctor. The rate of Arkansans reporting a personal doctor increased by 5.3pp (95% CI 1.7 to 8.8) during 2014-2016. No statistically significant change was observed during 2017-2021 (2.8pp; 95% CI −0.3 to 5.9). In our DID model, no statistically significant differences were observed in rates of having a personal doctor in Arkansas relative to traditional expansion states during 2014-2016, 2017-2021, or the full post-expansion period (+1.3pp, 95% CI −2.2 to 4.8).

### Avoided care due to cost in the previous 12 months

Throughout the study period, Arkansans were more likely to avoid care due to cost than residents of TE states. In unadjusted analyses, 8.8pp more Arkansans avoided care due to cost compared to TE state residents in 2011. These differences remained relatively stable over time, with 7.9pp fewer Arkansans avoiding care due to costs relative to TE states in 2021. The rate of avoided care among Arkansans declined by 10.3pp (95% CI −13.7 to −6.9) during 2014-2016 and declined further by 4.1pp (95% CI −7.4 to −0.9) from 2017-2021. In our DID model, no statistically significant differences were observed in rates of avoided care due to cost in Arkansas relative to traditional expansion states during 2014-2016, 2017-2021, or the full post-expansion period (−0.1pp, 95% CI −3.1 to 2.9).

### Receipt of routine checkup in the past 12 months

In 2011, Arkansans were 13.4pp less likely to report an annual checkup compared to TE states. By 2021, this difference declined to 1.4pp. The rate of Arkansans receiving routine checkups increased by 10.0pp (95% CI 6.5 to 13.6) during 2014-2016 and increased further by 9.5pp (95% CI 6.2 to 12.8) during 2017-2021. In our DID model, Arkansas' expansion was associated with a 4.6pp (95% CI 0.6 to 8.5) increase in the likelihood of receiving a routine checkup during 2014-2016, with no additional significant changes experienced during 2017-2021. Overall, rates of having a routine checkup in Arkansas increased by an additional 6.1pp (95% CI 2.6 to 9.5) compared to TE states during the entire outcome period.

### Received a flu shot in the past 12 months

Unadjusted rates of flu vaccination slightly increased in Arkansas and increased in TE states during the study period. Flu vaccination rates in Arkansas were 1.2pp and 5.3pp lower than TE states in 2011 and 2021, respectively. Flu vaccination rates among Arkansans increased significantly by 4.6pp (95% CI 1.1 to 8.1) during 2014-2016, with no additional significant changes experienced during 2017-2021 (−2.1pp, 95% CI −5.2 to 1.0). In our DID model, no statistically significant change was observed during 2014-2016, 2017-2021, or the full post-expansion period (+1.3pp, 95% CI −2.2 to 4.7).

### Comparison to non-expansion states

Next, we compared Arkansas to states that never expanded Medicaid (Supplementary Appendix A8). Compared to non-expansion states, Arkansas' expansion was associated with a 21.9pp increase in insurance coverage (95% CI 21.6 to 22.9), a 5.6pp increase in rates of having a personal doctor (95% CI 5.0 to 6.3), a 5.3pp decrease in avoided care due to cost (95% CI −6.9 to −3.7), and an 11.1pp increase in rates of having a routine checkup (95% CI 9.2 to 13.0) from baseline to the waiver extension period. We did not observe differential changes in flu vaccination rates.

### Sensitivity analyses

Our findings were robust to the exclusion of 2020-2021 data, with no substantive changes to our point estimates or confidence intervals (Supplementary Appendix A9). The treatment and control groups were more similar after the IPTW procedure (Supplementary Appendix A10); SMDs declined markedly for household income, race, and veteran status. However, incorporation of IPTW weights into our regression models did not substantively change our point estimates or confidence intervals (Supplementary Appendix A11).

Our DID estimates were consistent in direction but varied in magnitude when Arkansas was compared with the full set of 15 traditional expansion states. For the full post-expansion period, the estimated difference in insurance coverage more than doubled (+9.6pp, 95% CI 7.1 to 12.0), and the estimate for receipt of a routine checkup modestly increased (+7.8pp, 95% CI 6.3 to 9.2), while estimates for other outcomes remained statistically insignificant. However, this broader comparison group differs substantially from Arkansas in demographic composition, particularly with respect to racial and ethnic makeup and the proportion of Hispanic respondents (Supplementary Appendix A12). Prior research has documented smaller coverage gains from Medicaid expansion among Hispanic immigrant populations,^39-41^ likely reflecting immigration-related eligibility restrictions or concerns over public charge laws.^42-45^ As a result, comparisons using this counterfactual may overstate differences in coverage gains relative to Arkansas.

Lastly, we reanalyzed our data without imputation of missing covariates and our findings were consistent (Supplementary Appendix A13).

## Discussion

Arkansas' Medicaid expansion waiver is the subject of heightened policy and research interest due to several unique policy components. These include premium subsidies for enrollees to purchase insurance plans through the state's individual insurance marketplace, temporary imposition of work requirements, and requirements for enrollees to make premium payments. The policy relevance of our findings is heightened by the 2025 reconciliation bill, which established national Medicaid work requirements and shifts attention toward how other design features of expansion models may affect access to care. Work requirements applied to adults aged 30-49 for a brief portion of our study period. By contrast, other waiver components such as premium assistance through private Marketplace plans remain relevant for state and federal policy design. Our results suggest that low-income Arkansans experienced large and durable gains in insurance coverage and preventive service utilization, even in the presence of modest cost-sharing provisions. Policymakers should weigh these findings carefully as they consider additional reforms.

While we are unable to directly observe source of insurance coverage prior to 2021 in the BRFSS, our results comport with CMS' estimates of Medicaid and CHIP enrollment that showed sizeable gains in coverage.^46^ Arkansas had 630 196 individuals enrolled in Medicaid or CHIP (across all ages) in December 2013.^47^ Enrollment rose to 857 853 by the end of our study period, an increase in enrollment by 36%.^46^ We expand upon these previous descriptive findings, providing a rigorous comparison of Arkansas and TE states. Our results compare favorably with previously reported results for traditional Medicaid expansions under the ACA.^48,49,50^

Arkansas had substantially lower baseline access and stricter pre-ACA eligibility, which may explain the magnitude of the observed gains. Childless adults were ineligible for Medicaid prior to expansion in both Arkansas and TE states. However, Arkansas' Medicaid income eligibility limits were much lower for parents who had to earn no more than 16% FPL to receive full Medicaid benefits in 2013.^38^ As a result, the larger absolute coverage gains observed in Arkansas likely reflect convergence from a lower baseline and the resolution of greater unmet need, rather than evidence that the market-based expansion model outperformed traditional Medicaid expansions.

Although our study cannot disentangle the effects of individual waiver components, prior evidence helps interpret the patterns we observe. In particular, Arkansas' use of Marketplace QHPs may have supported access by expanding provider networks and improving enrollee experiences,^16-19^ which could help explain the gains observed here. At the same time, our findings should not be interpreted as evidence that all waiver features were benign. Arkansas's work requirements were in effect only briefly, yet existing evidence shows that such requirements reduce coverage and access.^5,14,15^ This distinction between market-based enrollment features that may facilitate access, vs cost-sharing or work requirements that may create barriers, is especially relevant to current policy debates.

While Arkansas differs from comparison states on certain demographic and pre-expansion policy dimensions, our objective was not to construct an ideal counterfactual but to assess whether changes in access under Arkansas' waiver-based Medicaid expansion were comparable in magnitude to those observed in traditional expansions—a policy-relevant question for states currently considering alternative approaches to Medicaid expansion. Our findings are also relevant for current federal and state-level debates around Medicaid waiver design. Although we are unable to isolate the effects of individual policy components within Arkansas' waiver, our results suggest that market-based expansion models may generate meaningful access gains and reduce the harms of uninsurance. These lessons remain instructive for federal policy debates, even though state-level 1115 work requirements are no longer permissible under current law.

### Limitations

There are several limitations of our study. First, our outcomes are self-reported, though prior studies demonstrate high reliability and validity.^33^ Second, median BRFSS response rate ranges from 45.2% to 54.6% during our study period,^51^ which compares favorably with most household surveys.^21^ Third, income is reported categorically, so our FPL measure is a proxy, though this is a validated approach.^52^ Fourth, we are unable to determine the effects of any specific components of Arkansas' waiver. We cannot assess the effects of Arkansas' use of premium assistance separately from premium requirements or the work and community engagement component. Fifth, prior to 2021 the BRFSS does not identify source of insurance coverage, which prevents us from distinguishing Medicaid enrollment from other forms of coverage. Because Arkansas's expansion relied on Marketplace QHPs, respondents in Arkansas may have been more likely to report having insurance than those enrolled in traditional Medicaid, potentially leading to modest differential measurement of insurance coverage. Nevertheless, this outcome has been widely used to assess changes in coverage following Medicaid expansion. Sixth, our data include only 3 pre-intervention years which limit our ability to draw strong conclusions about pre-treatment trends. The BRFSS included cell phones in their sampling starting in 2011, limiting data comparability with previous years. Lastly, while the Medicaid adult expansion population includes adults aged 19-64, BRFSS age variables begin at age 18, resulting in a slight age range mismatch.

### Conclusions

Our findings suggest that Arkansas's market-based Medicaid expansion was associated with substantial gains from a low baseline, resulting in post-expansion levels of insurance coverage and preventive service utilization comparable to traditional expansion states. These results underscore that alternative expansion models may reduce pre-existing coverage gaps, rather than necessarily outperform traditional Medicaid designs. State approaches to Medicaid expansion that leverage market-driven options might represent a viable alternative to improve access to care for low-income adults. Ten states have yet to expand Medicaid income eligibility despite new financial incentives provided by the American Rescue Plan Act of 2021.^53^ Despite its popularity with voters, it is impossible or highly unlikely to expand Medicaid through ballot initiatives in these holdout states—state legislatures must decide.^54,55^ Arkansas' market-driven approach may be more attractive to conservative policymakers compared to traditional state Medicaid expansions. As such, examining the real-world effects of these waiver features is critical for informing ongoing policy debates, particularly in states that have yet to expand Medicaid or are exploring alternative models to do so.

## Supplementary Material

qxag045_Supplementary_Data
