# Supplementary material for: Health access and prevention under Arkansas' market-based Medicaid expansion
Source: Health Aff Sch. 2026 Feb 25;4(3):qxag045. doi: 10.1093/haschl/qxag045 (PMC13008014; doi:10.1093/haschl/qxag045)
Supplement: qxag045_Supplementary_Data [file qxag045_supplementary_data.zip › AR Medicaid Expansion Appendix 2026 02 02.docx]

**SUPPLEMENTAL APPENDIX**

**Methodological Supplement**

**Federal Poverty Level (FPL) Imputation Procedure:**

Following the work of Sommers et al. (2015), we used an imputation procedure to convert the household income data contained in the Behavioral Risk Factor Surveillance System (BRFSS) to FPL percentage. We first converted each of the survey’s income categories into a dollar term using the midpoint of the category range (e.g., $30,000 for people reporting household income between $25,000 and $35,000). We then used household size and the U.S. federal poverty guidelines to convert income into a percentage of FPL.

**Imputation**

We used multiple imputations using additive regression (10 imputations), bootstrapping, and predictive mean matching to replace missing covariate values and reduce the potential for non-response bias. When there were ties for the most often imputed value, one was selected at random. All covariates were used in the imputation model. Following best practices for non-linear regression models, outcomes were not imputed.

**Main Regression Analyses**

As described in the main text, all regression models were estimated using logistic regressions and incorporated BRFSS sampling weights. In sensitivity analyses, models were re-estimated using logistic regression. All vectors are bolded, while scalars are not. All analyses were conducted using R Statistical Software 4.4.2 (R Foundation for Statistical Computing, Vienna, Austria).

We first estimated simple models to determine adjusted annual trends in outcomes:

1. $Y_{it}=\alpha_{0}+\beta_{t}+\varepsilon_{it}$

Where *i* represents individuals and *t* represents time periods. $\beta_{t}$ are indicator variables for whether the respondent was interviewed in each year 2011-2021. Lastly, $\varepsilon_{it}$ is the error term. Models were stratified by whether the respondent was a resident in Arkansas or a traditional expansion (TE) state.

We then estimated stratified covariate-adjusted regression models to determine adjusted changes in outcomes:

1. $Y_{it}=\alpha_{s}+\beta_{1}{Early}_{t}+\beta_{2}{Late}_{t}+\delta X_{it}+\varepsilon_{it}$

With the baseline period January 2011 – December 2013 set as the reference period. ${Early}_{t}$ is a binary indicator taking on a value of one if the survey was conducted 2014-2016, 0 otherwise. ${Late}_{t}$ is a binary indicator taking on a value of one if the survey was conducted 2017-2021, 0 otherwise. Thus, the coefficient $\beta_{1}$ reflects changes in outcomes during the early expansion period (January 2014 – December 2016) and the coefficient $\beta_{2}$ reflects changes in outcomes during the early expansion period (January 2017 – December 2021). The vector $\delta X_{it}$ represents the coefficients for study covariates including survey month, age, marital status, educational attainment, race/ethnicity, employment status, household size, veteran status, household income, home ownership, presence of children in the household, and whether the survey was completed by a land line or mobile phone. The vector $\alpha_{s}$ represents state fixed effects. Models were again stratified by whether the respondent was a resident in Arkansas or a TE state.

We then estimated difference-in-differences (DID) models to determine whether Arkansas’ Medicaid expansion status mediated the observed changes in outcomes:

1. $Y_{ist}=\alpha_{s}+\beta_{ist}[{Arkansas}_{s}\times\left( {Early}_{t}+{Late}_{t}+X_{it} \right)]+\varepsilon_{ist}$

Where ${Arkansas}_{s}$ is a binary variable taking on a value of one if the respondent was a resident of Arkansas, zero otherwise. The coefficients for the interaction terms ${Arkansas}_{s}\times{Early}_{t}$and ${Arkansas}_{s}\times{Late}_{t}$ thus represent our DID estimates for the differential changes in outcomes between Arkansas and TE states during 2014-2016 and 2017-2021, respectively. Covariates are also interacted with treatment status, ${Arkansas}_{s}$, to ensure comparability between our DID and stratified covariate-adjusted regression models. All DID models used robust standard errors clustered at the state level.

We re-estimated our models where the binary variable ${Early}_{t}$ took on a value of 1 if the survey was conducted 2014-2021. In these specifications, the coefficient for the interaction ${Arkansas}_{s}\times{Late}_{t}$ indicated whether the changes in outcomes during the late expansion period were significantly different from the changes observed during the early expansion period.

Lastly, we re-estimated the DID model with a single post period to estimate changes in Arkansas compared to TE states for the entire outcome period (2014-2021):

1. $Y_{ist}=\alpha_{s}+\beta_{ist}[{Arkansas}_{s}\times\left( {Post}_{t}+X_{it} \right)]+\varepsilon_{ist}$

**Parallel Trends Assumption**

We tested the parallel trends assumption by estimating logistic regression models with baseline data and including our covariates, a linear time trend, a treatment status indicator, and an interaction term between time and treatment:

1. $Y_{ist}=\alpha_{s}+{\beta_{ist}[Arkansas}_{s}\times({Time}_{t}+X_{it})]+\varepsilon_{ist}$

${Time}_{t}$ is measured in years (e.g., 2011 is 0, 2021 is 10). Thus, the coefficient for the interaction term ${Arkansas}_{s}\times{Time}_{t}$ indicates whether there were differential pre-trends in outcomes between Arkansas and TE states. Event studies, while commonly used as an alternative to test parallel trends, measure differences in pre-levels during each time period versus differences in pre-trends. We include event study model results for completeness; their specifications are the same as (4) except ${Time}_{t}$ is now a vector of binary variables for each year with 2013 set as the reference period.

| **Appendix A1. List of Study Outcomes and Covariates** | | | | |  |
| --- | --- | --- | --- | --- | --- |
| **Outcome Name** | **BRFSS Variable Name(s) and Years** | **Survey Question** | **Response Format** | | **Item-Level Missingness** |
| **Outcome Variables** | | | |  |  |
| Has Insurance Coverage^1^ | HLTHPLN1: BRFSS Years 2011 – 2020  PRIMINSR: BRFSS Years 2021 | “Do you have any kind of health care coverage, including health insurance, prepaid plans such as HMOs, or government plans such as Medicare, or Indian Health Service?” | Coded 1 if yes, 0 if no |  | 0.8% |
| Has a Personal Doctor | PERSDOC2: BRFSS Years 2011 – 2020  PERSDOC3: BRFSS Years 2021 | “Do you have one person (or a group of doctors) that you think of as your personal health care provider?” | Coded 1 if yes, 0 if no |  | 0.5% |
| Flu Vaccine | FLUSHOT5: BRFSS Years 2011 – 2012  FLUSHOT6: BRFSS Years 2013 – 2018  FLUSHOT7: BRFSS Years 2019 – 2021 | “During the past 12 months, have you had either flu vaccine that was sprayed in your nose or flu shot injected into your arm?” | Coded 1 if yes, 0 if no |  | 7.3% |
| Avoided Care Due to Cost | MEDCOST: BRFSS Years 2011 – 2020  MEDCOST: BRFSS Years 2021 | "Was there a time in the past 12 months when you needed to see a doctor but could not because you could not afford it?” | Coded 1 if yes, 0 if no |  | 0.3% |
| Last Routine Checkup^2^ | CHECKUP1 | "About how long has it been since you last visited a doctor for a routine checkup? [A routine checkup is a general physical exam, not an exam for a specific injury, illness, or condition.]” | Within past year,  within past 2 years,  within past 5 years,  5 or more years ago. Coded as 1 if within the past year, 0 otherwise |  | 1.4% |
| **Covariates** | | | |  |  |
| Age | _AGEG5YR | “What is your age?” | 18-24, 25-29, 30-34, 35-39, 40-44, 45-49, 50-54, 55-59, 60-64 |  | 0.9% |
| Educational Attainment | _EDUCAG | “What is the highest year or grade of school you have completed?” | Coded as completed college (4-year degree or more), some college (less than a 4-year degree), no college |  | 0.2% |
| Preferred Race | _RACE_G: BRFSS Years 2011 – 2012  _RACE_G1: BRFSS Years 2013 – 20  RACEPRV: BRFSS Years 2020 – 2021 | “Which one or more of the following would you say is your race?” | White Non-Hispanic, Black Non-Hispanic, Asian Non-Hispanic, American Indian/Alaskan Native Non-Hispanic, Hispanic, Other race Non-Hispanic |  | 1.2% |
| Employment Status | EMPLOY: BRFSS Years 2011 – 2012  EMPLOY1: BRFSS Years 2013 – 2021 | “Are you currently employed for wages, self-employed, out of work for 1 year or more, out of work for less than 1 year, a homemaker, a student, retired, or unable to work?” | Coded as currently employed, unemployed, or not in the labor force (homemaker, student, retired, unable to work) |  | 0.6% |
| Household Size | _CHLDCNT and _NUMADULT | Children: “How many children less than 18 years of age live in your household?”  Adults: “Excluding adults living away from home, such as students away at college, how many members of your household, including yourself, are 18 years of age or older?” | Sum of total children and adults in household |  | 0.0% |
| Veteran Status | VETERAN3 | “Have you ever served on active duty in the United States Armed Forces, either in the regular military or in a National Guard or military reserve unit?” | Coded 1 if yes, 0 if no |  | 0.1% |
| Sex | SEX: BRFSS Years 2011 – 2017  SEX1: BRFSS Years 2018 – 2021 | “Are you male or female?” | Coded 1 if male, 0 if female |  | 0.0% |
| Income^3^ | _INCOMG: BRFSS Years 2011 – 2020  _INCOMG1: BRFSS Years 2021 | “Is your annual household income from all sources…” | Less than $10,000, Less than $15,000 ($10,000 to less than $15,000), Less than $20,000 ($15,000 to less than $20,000), Less than $25,000 ($20,000 to less than $25,000), Less than $35,000 ($25,000 to less than $35,000) Less than $50,000 ($35,000 to less than $50,000), Less than $75,000? ($50,000 to less than $75,000), $75,000 or more. |  | 0.0% |
| Indicator for landline or cell phone | LANDLINE | “Is this a cell telephone?” | Coded 1 if yes, 0 if no |  | 0.0% |
| Homeownership status | RENTHOM1 | “Do you own or rent your home?” | Coded1 if the respondent owns their home, 0 otherwise |  | 0.8% |

**Source:** Authors' analysis of data from low-income adult respondents to the 2011-2021 Behavioral Risk Factor Surveillance System (BRFSS). **Notes:** ^1^Starting in 2021, this question was modified to “What is the current primary source of your health insurance?” Respondents could identify source of insurance (e.g., Medicaid, Medicare, employer) or state they were uninsured. Responses were binary coded to insured/uninsured for consistency with earlier years. ^2^This question was binary coded to indicate whether the respondent received a routine checkup within the past year. ^3^Starting in 2021, the following categories were added, and the $75,000 or more category was removed: less than $100,000? ($75,000 to less than $100,000), less than $150,000? ($100,000 to less than $150,000), less than $200,000? ($150,000 to less than $200,000), and $200,000 or more.

**Appendix A2. Event study estimates for the effects of Arkansas’ Medicaid expansion on insurance coverage**

**
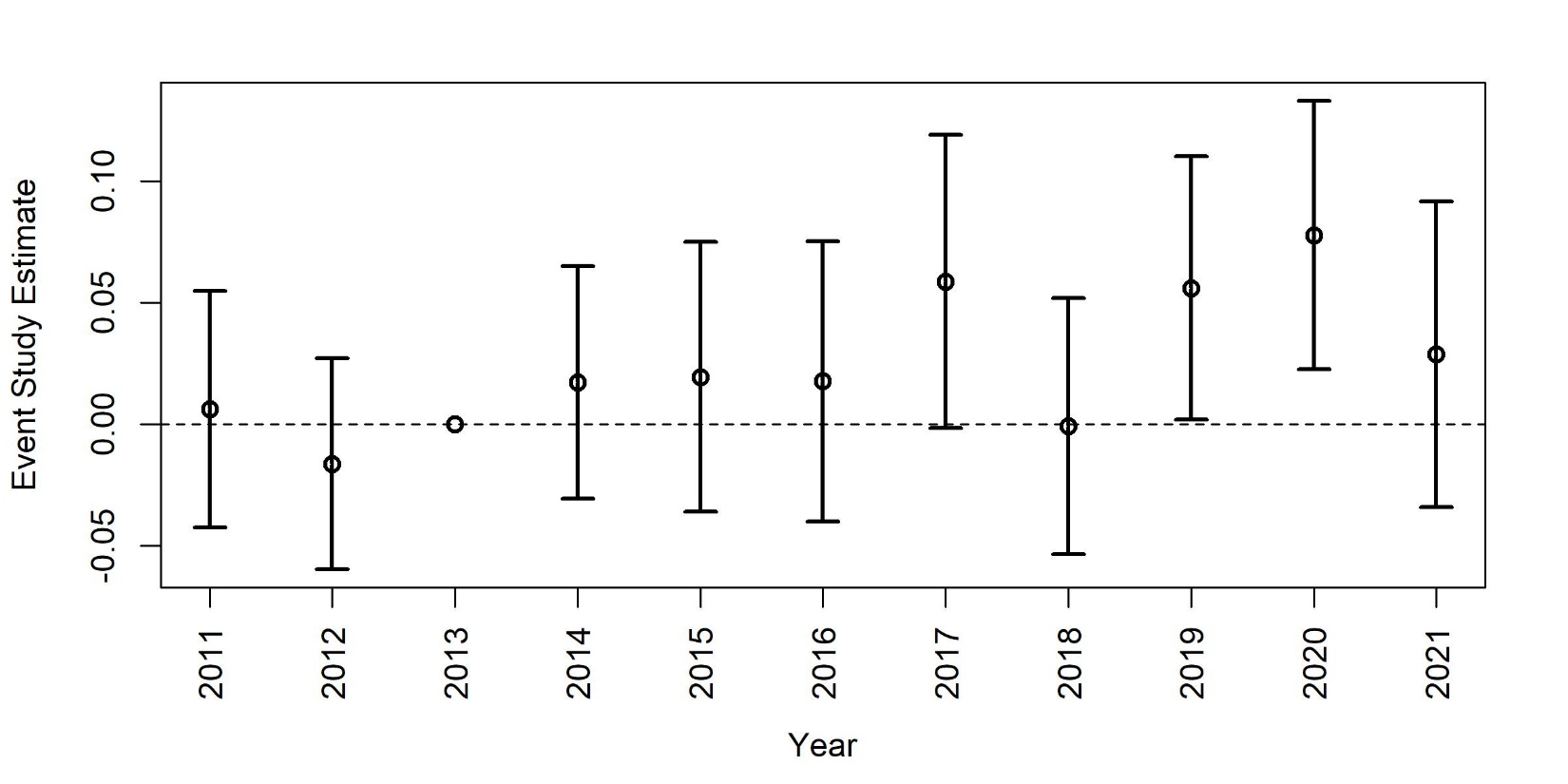
**

**Source:** Authors' analysis of data from low-income adult respondents to the 2011-2021 Behavioral Risk Factor Surveillance System (BRFSS). **Notes:** The figure displays regression-adjusted percentage-point changes in the likelihood of insurance coverage during each period, with 2013 as the reference year. The model incorporated BRFSS sampling weights with standard errors clustered at the state level, and was adjusted for respondent age, marital status, education, employment status, household size, veteran status, sex, household income, homeownership status, survey month, and whether the survey was conducted via landline or cell phone.

**Appendix A3. Event study estimates for the effects of Arkansas’ Medicaid expansion on having a personal doctor**

**
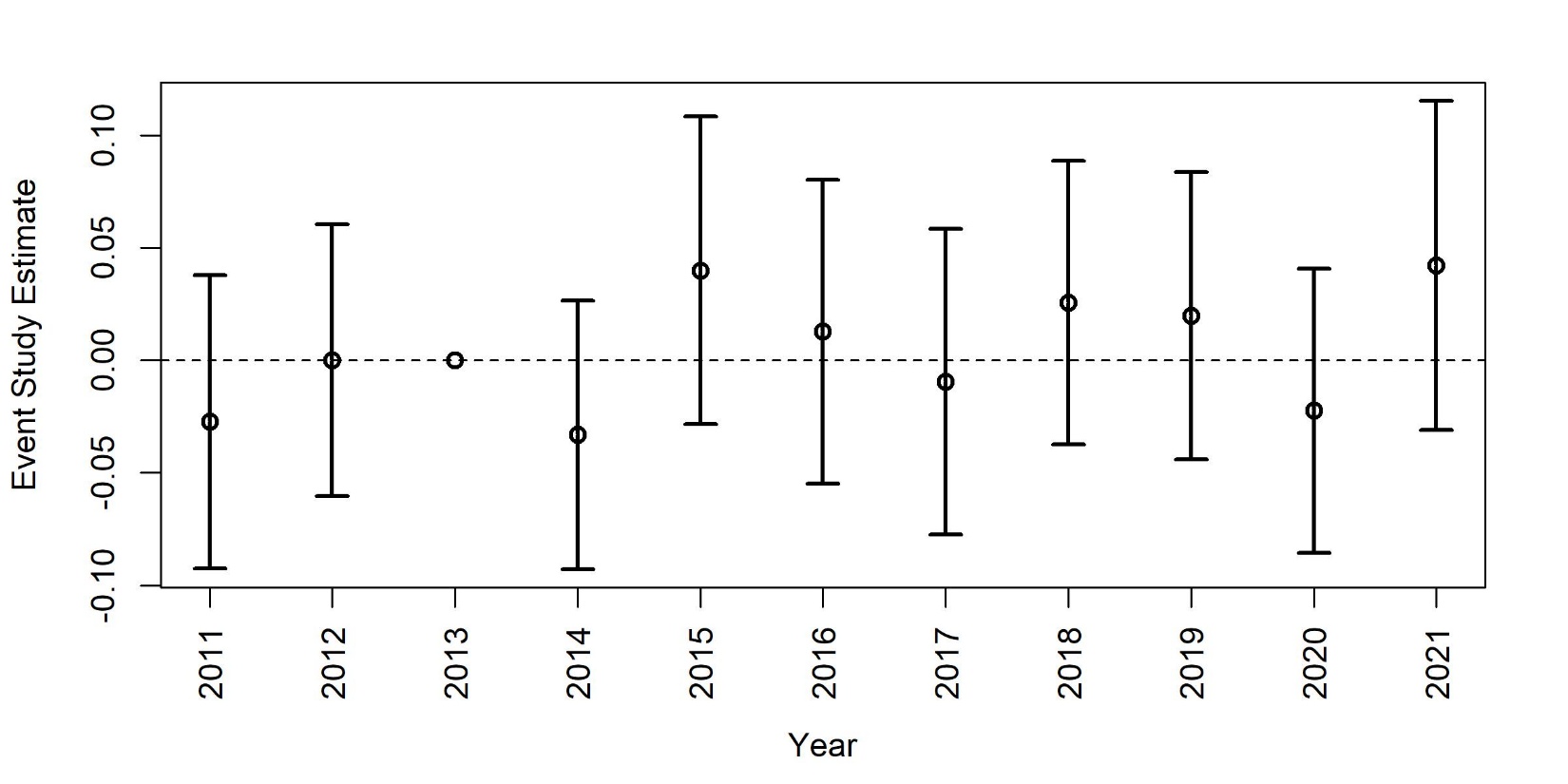
**

**Source:** Authors' analysis of data from low-income adult respondents to the 2011-2021 Behavioral Risk Factor Surveillance System (BRFSS). **Notes:** The figure displays regression-adjusted percentage-point changes in the likelihood of having a personal doctor during each period, with 2013 as the reference year. The model incorporated BRFSS sampling weights with standard errors clustered at the state level, and was adjusted for respondent age, marital status, education, employment status, household size, veteran status, sex, household income, homeownership status, survey month, and whether the survey was conducted via landline or cell phone.

**Appendix A4. Event study estimates for the effects of Arkansas’ Medicaid expansion on avoided care due to cost**

**
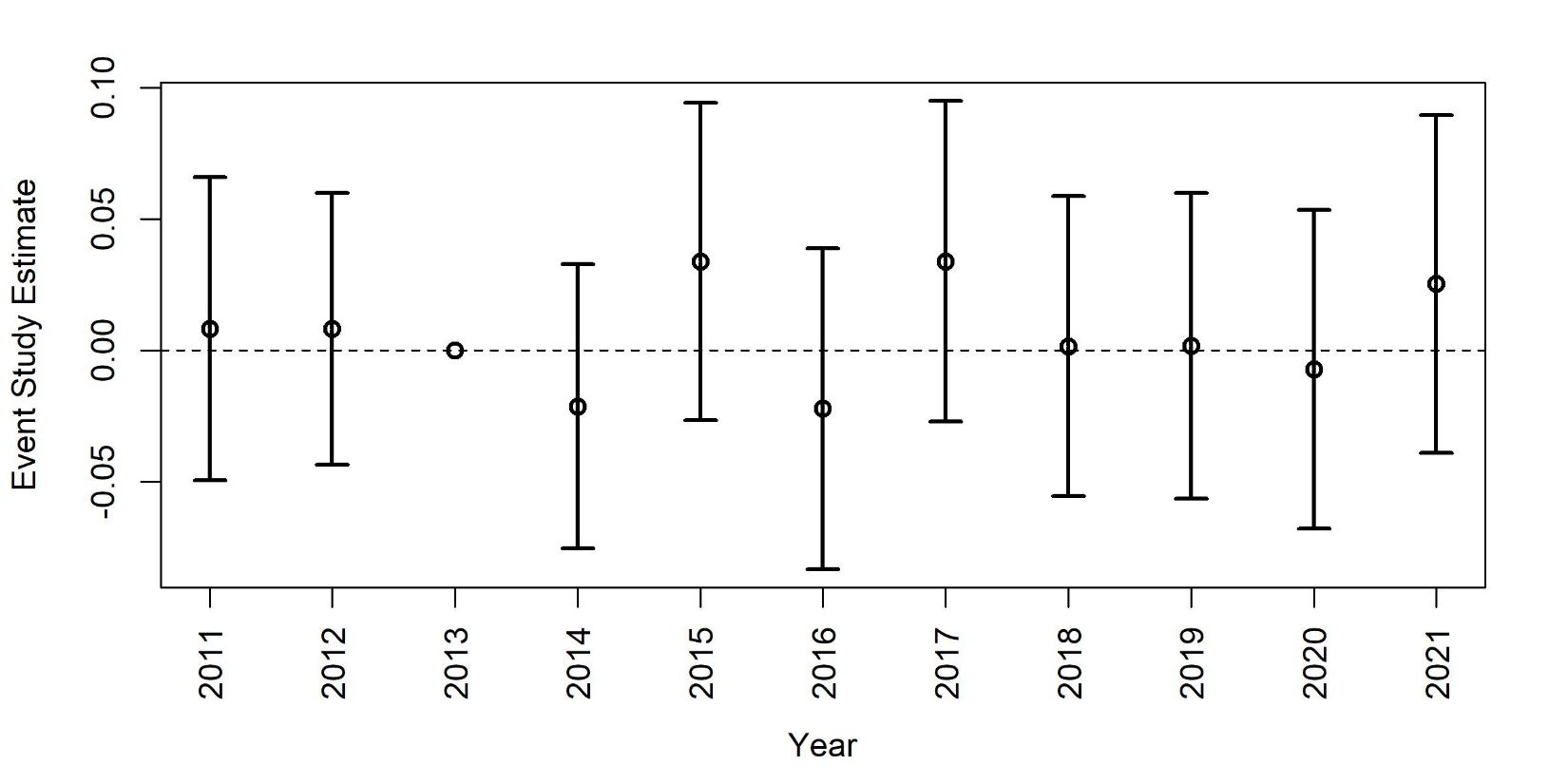
**

**Source:** Authors' analysis of data from low-income adult respondents to the 2011-2021 Behavioral Risk Factor Surveillance System (BRFSS). **Notes:** The figure displays regression-adjusted percentage-point changes in the likelihood of avoided care due to cost during each period, with 2013 as the reference year. The model incorporated BRFSS sampling weights with standard errors clustered at the state level, and was adjusted for respondent age, marital status, education, employment status, household size, veteran status, sex, household income, homeownership status, survey month, and whether the survey was conducted via landline or cell phone.

**Appendix A5. Event study estimates for the effects of Arkansas’ Medicaid expansion on routine checkups**

**
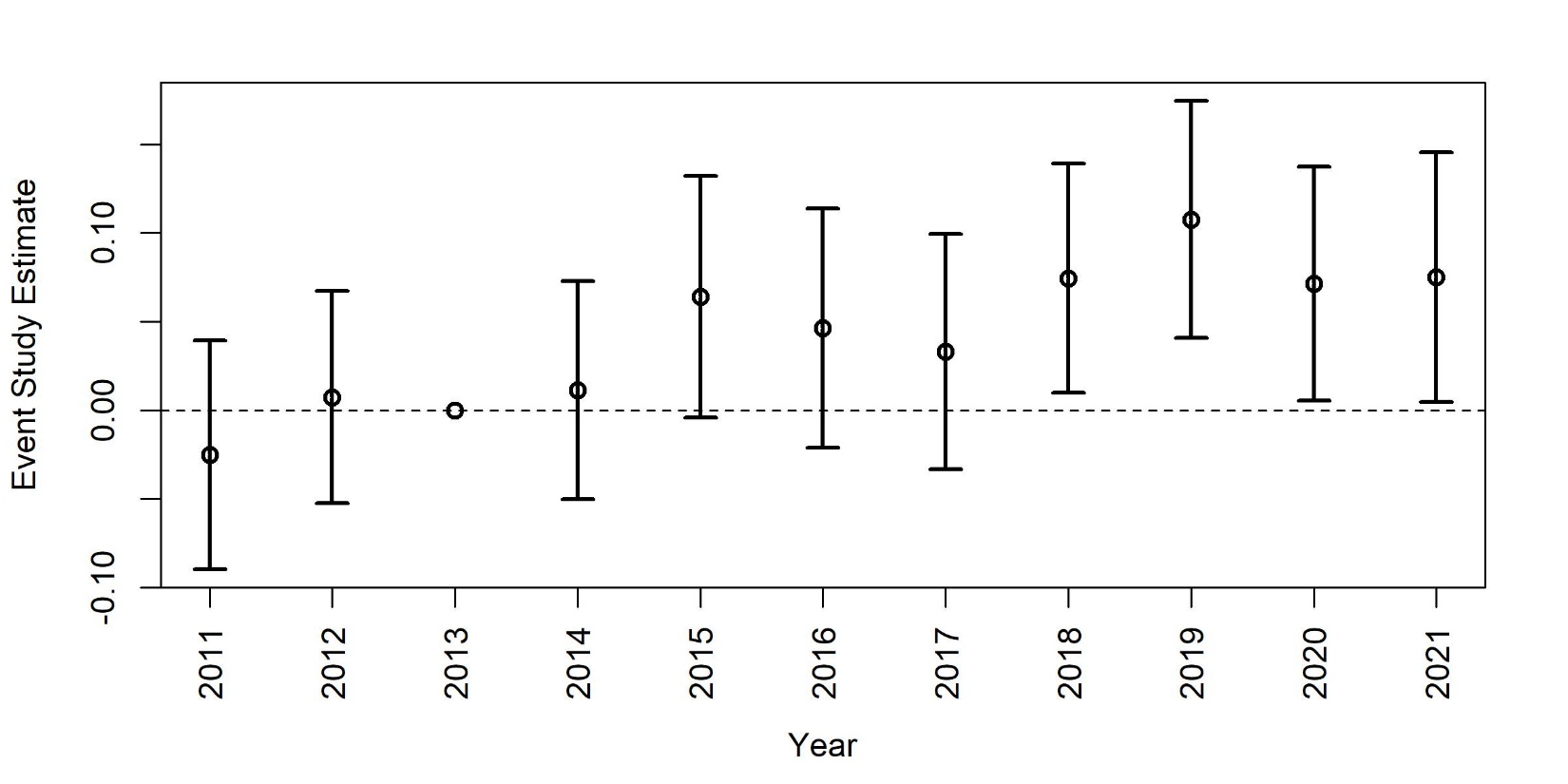
**

**Source:** Authors' analysis of data from low-income adult respondents to the 2011-2021 Behavioral Risk Factor Surveillance System (BRFSS). **Notes:** The figure displays regression-adjusted percentage-point changes in the likelihood of receiving annual checkups during each period, with 2013 as the reference year. The model incorporated BRFSS sampling weights with standard errors clustered at the state level, and was adjusted for respondent age, marital status, education, employment status, household size, veteran status, sex, household income, homeownership status, survey month, and whether the survey was conducted via landline or cell phone.

**Appendix A6. Event study estimates for the effects of Arkansas’ Medicaid expansion on flu vaccination**


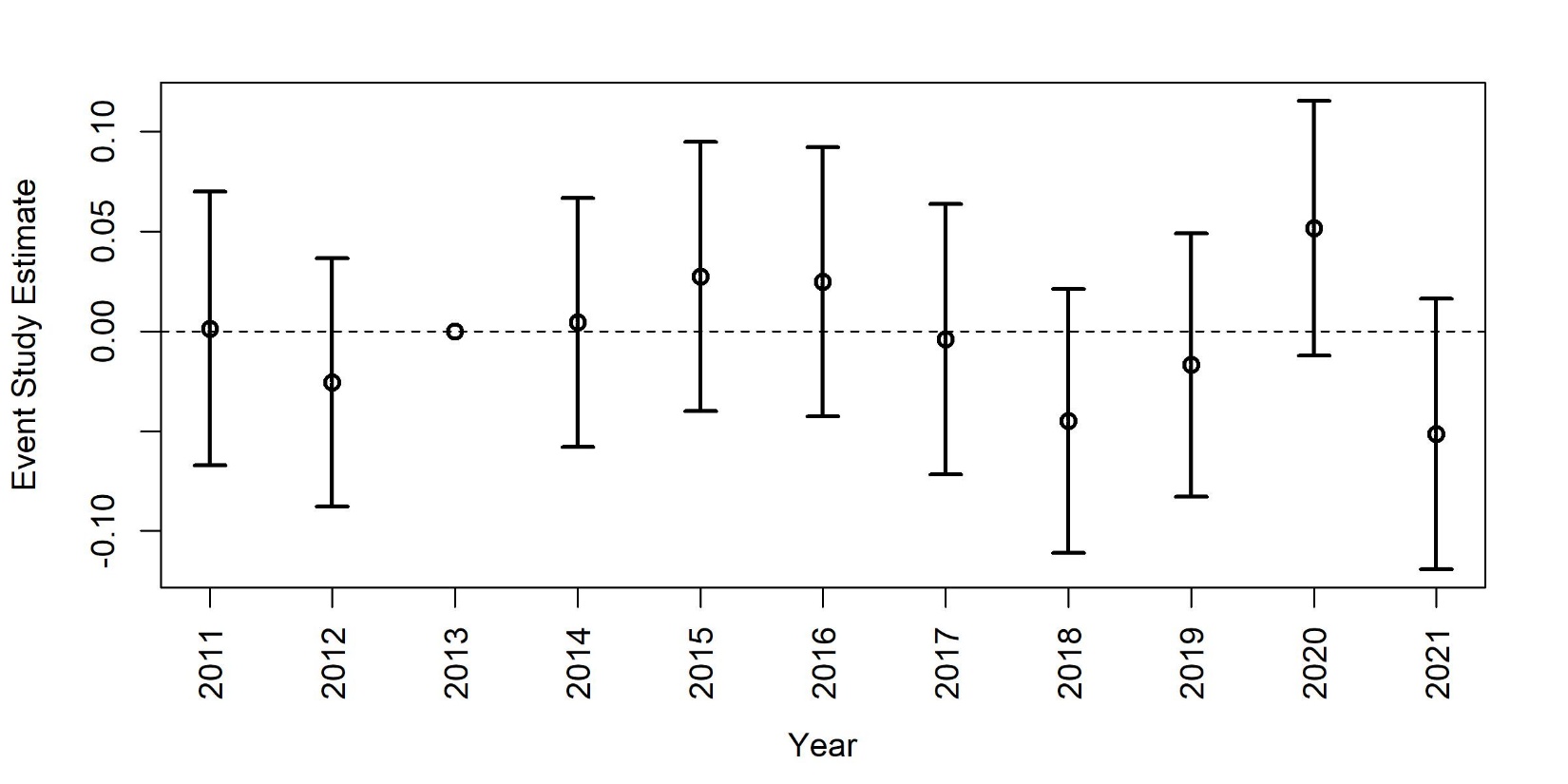


**Source:** Authors' analysis of data from low-income adult respondents to the 2011-2021 Behavioral Risk Factor Surveillance System (BRFSS). **Notes:** The figure displays regression-adjusted percentage-point changes in the likelihood of flu vaccination during each period, with 2013 as the reference year. The model incorporated BRFSS sampling weights with standard errors clustered at the state level, and was adjusted for respondent age, marital status, education, employment status, household size, veteran status, sex, household income, homeownership status, survey month, and whether the survey was conducted via landline or cell phone.

| **Appendix A7. Unadjusted rates of health care access for Arkansas and three traditional expansion states, 2011-2021** | | | | | | | | | | | | | |
| --- | --- | --- | --- | --- | --- | --- | --- | --- | --- | --- | --- | --- | --- |
| **Outcome** | **Arkansas/ TE^1^** | **Sample Size 2011-2021** | **Unadjusted rates** | | | | | | | | | | |
|  |  |  | **2011** | **2012** | **2013** | **2014** | **2015** | **2016** | **2017** | **2018** | **2019** | **2020** | **2021** |
| Have Health Care Coverage | Arkansas | 9,820 | 52.4 | 48.0 | 52.6 | 70.8 | 77.3 | 82.1 | 84.6 | 79.4 | 78.5 | 82.3 | 83.7 |
|  | TE | 52,218 | 66.5 | 65.3 | 67.3 | 79.4 | 84.6 | 88.0 | 87.2 | 87.9 | 83.9 | 84.2 | 88.8 |
| Have Personal Doctor | Arkansas | 9,842 | 62.6 | 65.6 | 66.2 | 65.6 | 73.5 | 72.7 | 72.4 | 73.0 | 71.5 | 67.1 | 80.1 |
|  | TE | 52,372 | 68.9 | 69.5 | 69.8 | 69.5 | 71.5 | 72.4 | 74.3 | 71.4 | 73.0 | 70.7 | 79.1 |
| Avoided Care Due to Cost | Arkansas | 9,731 | 41.8 | 44.6 | 42.1 | 34.1 | 31.3 | 26.0 | 28.7 | 25.7 | 29.0 | 21.9 | 26.3 |
|  | TE | 51,669 | 33.0 | 34.2 | 33.2 | 29.3 | 21.1 | 21.2 | 19.9 | 19.1 | 21.6 | 17.0 | 18.4 |
| Last Routine Checkup | Arkansas | 9,849 | 48.4 | 52.2 | 52.0 | 55.8 | 62.3 | 65.7 | 62.3 | 71.5 | 75.5 | 70.8 | 70.7 |
|  | TE | 52,378 | 61.8 | 61.5 | 63.3 | 63.6 | 64.3 | 68.9 | 68.1 | 72.4 | 74.5 | 72.2 | 72.1 |
| Flu Vaccine | Arkansas | 9,135 | 25.8 | 24.8 | 29.3 | 30.0 | 32.9 | 30.6 | 31.2 | 22.2 | 27.6 | 37.9 | 24.9 |
|  | TE | 49,846 | 27.0 | 28.3 | 30.1 | 29.9 | 29.9 | 28.0 | 32.9 | 26.2 | 31.1 | 32.2 | 30.2 |
| **Source:** Authors' analysis of data from low-income adult respondents to the 2011-2021 Behavioral Risk Factor Surveillance System (BRFSS). **Notes:** The table displays unadjusted outcome percentages during each period, accounting for BRFSS post-stratification weights. ^1^Traditional expansion (TE) states included Kentucky, Ohio, and West Virginia. | | | | | | | | | | | | | |

| **Appendix A8. Adjusted changes in health care access for Arkansas and non-expansion states, 2011-2021** | | | | | | | | | | | | |
| --- | --- | --- | --- | --- | --- | --- | --- | --- | --- | --- | --- | --- |
| **Outcome** | **Arkansas/ NE^1^** | **Estimated Differences** | | | | | | | | | | |
|  |  | **Initial Waiver vs. Baseline** | |  | **Waiver Extension vs. Baseline** | | |  | **Waiver Extension vs. Initial Waiver** | | | |
|  |  | **Estimate (95% CI)^2^** | **Difference (95% CI)^3^** |  | **Beta^2^** | **Difference^3^** |  |  | **Beta^2^** |  | **Difference^3^** |  |
| Have Insurance Coverage | Arkansas | 20.6*** (17.6, 23.6) | 15.3*** (14.6, 15.9) | | 26.5*** (23.6, 29.4) | 21.9*** (21.6, 22.9) | | | 5.9*** (2.8, 9.0) | | 6.6*** (5.7, 7.5) | |
|  | NE | 10.3*** (9.2, 11.5) | -- |  | 11.1*** (9.9, 12.3) | -- | |  | 0.8 (-0.3, 1.8) | | -- | |
| Have Personal Doctor | Arkansas | 5.4** (1.8, 9.0) | 3.5*** (1.9, 5.2) |  | 8.0*** (4.6, 11.4) | 5.6*** (5.0, 6.3) | |  | 2.6 (-0.5, 5.7) | | 2.1* (0.8, 3.4) | |
|  | NE | 2.7*** (1.6, 3.9) | -- |  | 3.6*** (2.4, 4.8) | -- | |  | 0.9 (-0.1, 1.9) | | -- | |
| Avoided Care Due to Cost | Arkansas | -10.5*** (-14.0, -7.1) | -4.2*** (-5.6, -2.8) | | -14.0*** (-17.3, -10.8) | -5.3*** (-6.9, -3.7) | |  | -3.5* (-6.8, -0.2) | | -1.1*** (-1.6, -0.6) | |
|  | NE | -6.8*** (-7.9, -5.7) | -- |  | -9.4*** (-10.5, -8.2) | -- | |  | -2.6*** (-3.6, -1.5) | | -- | |
| Last Routine Checkup | Arkansas | 10.2*** (6.6, 13.8) | 6.8*** (4.8, 8.7) | | 18.9*** (15.6, 22.3) | 11.1*** (9.2, 13.0) | |  | 8.8*** (5.4, 12.1) | | 4.3*** (3.1, 5.5) | |
|  | NE | 3.8*** (2.7, 4.9) | -- |  | 8.6*** (7.4, 9.8) | -- | |  | 4.8*** (3.8, 5.8) | | -- | |
| Flu Vaccine | Arkansas | 4.4* (0.9, 7.9) | 1.7 (-0.3, 3.7) |  | 2.1 (-1.3, 5.5) | 0.1 (-2.6, 2.8) | |  | -2.2 (-5.4, 0.9) | | -1.6 (-3.7, 0.5) | |
|  | NE | 2.5*** (1.4, 3.5) | -- |  | 1.9** (0.8, 3.1) | -- | |  | -0.5 (-1.5, 0.4) | | -- | |
| **Source:** Authors' analysis of data from low-income adult respondents to the 2011-2021 Behavioral Risk Factor Surveillance System (BRFSS). **Notes:** The table displays regression-adjusted percentage-point changes in outcomes during each period. Regression estimates are adjusted for respondent age, marital status, education, employment status, household size, veteran status, sex, household income, homeownership status, survey month, and whether the survey was conducted via landline or cell phone. The "Baseline" period includes 2011-2013, the "Initial Waiver" period includes 2014-2016, the "Waiver Extension" period includes 2017-2021. *p<0.05 **p<0.01 ***p<0.001. ^1^Non-expansion (NE) states included Alabama, Florida, Georgia, Kansas, Mississippi, South Carolina, Tennessee, Texas, Wisconsin, and Wyoming. ^2^Results from logistic regression models stratified by outcome and AR/TE group. ^3^Difference-in-difference estimates for changes in Arkansas and TE states over time. All models incorporated BRFSS sampling weights with standard errors clustered at the state level. | | | | | | | | | | | | |

| **Appendix A9. Adjusted changes in health care access for Arkansas and three traditional expansion states, 2011-2019 (excluding the COVID-19 period)** | | | | | | | | | | | | |
| --- | --- | --- | --- | --- | --- | --- | --- | --- | --- | --- | --- | --- |
| **Outcome** | **Arkansas/ TE^1^** | **Estimated Differences** | | | | | | | | | | |
|  |  | **Initial Waiver vs. Baseline** | |  | **Waiver Extension vs. Baseline** | | |  | **Waiver Extension vs. Initial Waiver** | | | |
|  |  | **Estimate (95% CI)^2^** | **Difference (95% CI)^3^** |  | **Beta^2^** | **Difference^3^** |  |  | **Beta^2^** |  | **Difference^3^** |  |
| Have Insurance Coverage | Arkansas | 21.5*** (18.5, 24.5) | 3.1 (-0.3, 6.4) |  | 26.9*** (23.6, 30.2) | 5.0** (1.3, 8.6) | |  | 5.4** (1.9, 8.9) | | 2.0 (-1.7, 5.6) | |
|  | TE | 16.0*** (14.4, 17.5) | -- |  | 18.8*** (17.2, 20.5) | -- | |  | 2.9** (1.1, 4.6) | | -- | |
| Have Personal Doctor | Arkansas | 5.6** (2.1, 9.2) | 1.3 (-2.7, 5.4) |  | 7.8*** (4.2, 11.5) | 2.1 (-2.1, 6.2) | |  | 2.2 (-1.2, 5.6) | | 0.8 (-3.1, 4.7) | |
|  | TE | 4.4*** (2.6, 6.2) | -- |  | 5.8*** (4.0, 7.7) | -- | |  | 1.5 (-0.3, 3.3) | | -- | |
| Avoided Care Due to Cost | Arkansas | -10.5*** (-14.0, -7.0) | -1.0 (-4.7, 2.6) |  | -13.1*** (-16.7, -9.5) | 0.9 (-2.9, 4.7) | |  | -2.6 (-6.2, 1.0) | | 1.9 (-1.8, 5.7) | |
|  | TE | -8.3*** (-9.9, -6.7) | -- |  | -12.6*** (-14.3, -10.8) | -- | |  | -4.2*** (-6.0, -2.5) | | -- | |
| Last Routine Checkup | Arkansas | 10.2*** (6.5, 13.9) | 4.5* (0.5, 8.6) |  | 18.9*** (15.2, 22.6) | 6.9** (2.7, 11.2) | |  | 8.7*** (5.0, 12.4) | | 2.4 (-1.7, 6.5) | |
|  | TE | 5.3*** (3.5, 7.1) | -- |  | 11.3*** (9.4, 13.2) | -- | |  | 6.0*** (4.1, 7.9) | | -- | |
| Flu Vaccine | Arkansas | 4.4* (0.9, 7.9) | 2.6 (-1.4, 6.5) |  | 0.6 (-3.1, 4.3) | -1.1 (-5.2, 3.1) | |  | -3.8* (-7.2, -0.4) | | -3.7 (-7.5, 0.2) | |
|  | TE | 1.9* (0.1, 3.6) | -- |  | 1.7 (-0.2, 3.5) | -- | |  | -0.2 (-1.9, 1.6) | | -- | |
| **Source:** Authors' analysis of data from low-income adult respondents to the 2011-2019 Behavioral Risk Factor Surveillance System (BRFSS). **Notes:** The table displays regression-adjusted percentage-point changes in outcomes during each period. Regression estimates are adjusted for respondent age, education, employment status, household size, veteran status, sex, household income, homeownership status, survey month, and whether the survey was conducted via landline or cell phone. The "Baseline" period includes 2011-2013, the "Initial Waiver" period includes 2014-2016, the "Waiver Extension" period includes 2017-2019. ^1^Traditional expansion (TE) states included Kentucky, Ohio, and West Virginia. ^2^Results from logistic regression models stratified by outcome and AR/Control group. ^3^Difference-in-difference estimates for changes in Arkansas and control states over time. All models incorporated BRFSS sampling weights with standard errors clustered at the state level. | | | | | | | | | | | | |

| **Appendix A10. Characteristics of the Study Sample after IPTW weighting (N=62,425)** | | | | | | | |
| --- | --- | --- | --- | --- | --- | --- | --- |
| Variable | | **Traditional expansion states,^1^ No. (weighted %)** | |  | **Arkansas, No. (weighted %)** | | **SMD** |
|  |  |  |  |  |  |  |  |
| Sex | |  |  |  |  |  |  |
|  | Female | 32,275 (55.6) | |  | 6,227 (53.5) | | 0.041 |
|  | Male | 20,260 (44.4) | |  | 3,663 (46.5) | |  |
| Marital Status | |  |  |  |  |  |  |
|  | Married | 18,195 (32.6) | |  | 3,529 (35.1) | | 0.054 |
|  | Unmarried | 34,340 (67.4) | |  | 6,361 (64.9) | |  |
| Household Income | |  |  |  |  |  | 0.044 |
|  | Less than $10,000 | 10,705 (19.5) | |  | 1,983 (19.3) | |  |
|  | $10,000 to $15,000 | 9,409 (15.3) | |  | 1,859 (16.6) | |  |
|  | $15,000 to $20,000 | 10,692 (19.4) | |  | 2,188 (19.7) | |  |
|  | $20,000 to $25,000 | 10,182 (20.6) | |  | 1,957 (19.5) | |  |
|  | $25,000 to $35,000 | 6,855 (14.7) | |  | 1,200 (14.9) | |  |
|  | $35,000 + | 4,692 (10.6) | |  | 703 (10.1) | |  |
| Race | |  |  |  |  |  |  |
|  | White | 43,318 (70.7) | |  | 6,215 (79.9) | | 0.241 |
|  | Black | 5,197 (19.0) | |  | 2,367 (10.9) | |  |
|  | Hispanic | 1,264 (5.5) | |  | 649 (5.3) | |  |
|  | Other | 1,326 (3.0) | |  | 342 (2.3) | |  |
|  | Multiracial | 1,430 (1.7) | |  | 317 (1.6) | |  |
| Age | |  |  |  |  |  |  |
|  | 18 to 24 | 4,521 (16.9) | |  | 754 (19.2) | | 0.094 |
|  | 25 to 34 | 8,451 (24.0) | |  | 1,535 (24.6) | |  |
|  | 35 to 44 | 9,563 (20.0) | |  | 1,815 (20.8) | |  |
|  | 45 to 54 | 12,546 (19.1) | |  | 2,367 (18.6) | |  |
|  | 55 to 64 | 17,454 (19.9) | |  | 3,419 (16.8) | |  |
| Education Group | |  |  |  |  |  |  |
|  | College grad | 46,051 (8.1) | |  | 8,686 (7.3) | | 0.030 |
|  | Not a college grad | 6,484 (91.9) | |  | 1,204 (92.7) | |  |
| Employment Group | |  |  |  |  |  |  |
|  | Unemployed | 6,107 (13.2) | |  | 1,107 (14.2) | | 0.030 |
|  | Employed | 20,248 (45.9) | |  | 3,887 (45.3) | |  |
|  | Not in labor force | 26,180 (40.9) | |  | 4,896 (40.5) | |  |
| Survey Modality | |  |  |  |  |  |  |
|  | Landline | 26,962 (36.7) | |  | 5,277 (36.5) | | 0.220 |
|  | Mobile phone | 25,573 (63.3) | |  | 4,613 (73.5) | |  |
| Veteran Status | |  |  |  |  |  |  |
|  | Veteran | 3,773 (6.8) | |  | 762 (7.2) | | 0.015 |
|  | Non-Veteran | 48,762 (93.2) | |  | 9,128 (92.8) | |  |

| **Source:** Authors' analysis of data from low-income adult respondents to the 2011-2021 Behavioral Risk Factor Surveillance System (BRFSS). **Notes:** Percentages incorporate BRFSS post-stratification weights and may not sum to 100 due to rounding. IPTW = inverse probability of treatment weighting; SMD = standardized mean differences. ^1^Traditional expansion states included Kentucky, Ohio, and West Virginia. |
| --- |

| **Appendix A11. Inverse probability of treatment weighted changes in health care access for Arkansas and three traditional expansion states, 2011-2021** | | | | | | | | | | | | |
| --- | --- | --- | --- | --- | --- | --- | --- | --- | --- | --- | --- | --- |
| **Outcome** | **Arkansas/ Control^1^** | **Estimated Differences** | | | | | | | | | | |
|  |  | **Initial Waiver vs. Baseline** | |  | **Waiver Extension vs. Baseline** | | |  | **Waiver Extension vs. Initial Waiver** | | | |
|  |  | **Estimate (95% CI)^2^** | **Difference (95% CI)^3^** |  | **Beta^2^** | **Difference^3^** |  |  | **Beta^2^** |  | **Difference^3^** |  |
| Have Insurance Coverage | Arkansas | 21.0*** (17.9, 24.1) | 3.5 (-0.1, 7.0) |  | 27.5*** (24.4, 30.6) | 6.6*** (3.1, 10.1) | |  | 6.5*** (3.2, 9.8) | | 3.2 (-0.3, 6.7) | |
|  | Control | 15.7*** (14.2, 17.2) | -- |  | 18.6*** (17.1, 20.0) | -- | |  | 2.9*** (1.3, 4.5) | | -- | |
| Have Personal Doctor | Arkansas | 7.1*** (3.4, 10.9) | 3.0 (-1.3, 7.2) |  | 9.1*** (5.6, 12.7) | 2.7 (-1.3, 6.7) | |  | 2.0 (-1.2, 5.3) | | -0.3 (-4.0, 3.3) | |
|  | Control | 4.2*** (2.4, 6.1) | -- |  | 6.6*** (4.9, 8.4) | -- | |  | 2.4** (0.7, 4.0) | | -- | |
| Avoided Care Due to Cost | Arkansas | -10.1*** (-13.6, -6.5) | -1.0 (-4.8, 2.9) |  | -15.1*** (-18.4, -11.7) | -0.1 (-3.7, 3.6) | |  | -5.0** (-8.4, -1.6) | | 0.9 (-2.7, 4.5) | |
|  | Control | -8.0*** (-9.6, -6.4) | -- |  | -13.3*** (-14.8, -11.7) | -- | |  | -5.3*** (-6.8, -3.7) | | -- | |
| Last Routine Checkup | Arkansas | 10.0*** (6.1, 13.9) | 4.5* (0.2, 8.7) |  | 20.2*** (16.6, 23.7) | 8.0*** (4.0, 12.0) | |  | 10.1*** (6.6, 13.7) | | 3.5 (-0.5, 7.4) | |
|  | Control | 5.1*** (3.2, 6.9) | -- |  | 11.2*** (9.5, 13.0) | -- | |  | 6.2*** (4.5, 7.9) | | -- | |
| Flu Vaccine | Arkansas | 4.7* (1.0, 8.3) | 2.7 (-1.4, 6.8) |  | 1.9 (-1.6, 5.4) | -0.6 (-4.6, 3.3) | |  | -2.7 (-6.0, 0.5) | | -3.3 (-7.0, 0.3) | |
|  | Control | 2.0* (0.2, 3.9) | -- |  | 2.6** (0.9, 4.3) | -- | |  | 0.5 (-1.1, 2.2) | | -- | |
| **Source:** Authors' analysis of data from low-income adult respondents to the 2011-2021 Behavioral Risk Factor Surveillance System (BRFSS). **Notes:** The table displays regression-adjusted percentage-point changes in outcomes during each period. Regression estimates are adjusted for respondent age, marital status, education, employment status, household size, veteran status, sex, household income, homeownership status, survey month, and whether the survey was conducted via landline or cell phone. The "Baseline" period includes 2011-2013, the "Initial Waiver" period includes 2014-2016, the "Waiver Extension" period includes 2017-2021. *p<0.05 **p<0.01 ***p<0.001. ^1^Traditional expansion (TE) states included Kentucky, Ohio, and West Virginia. ^2^Results from logistic regression models stratified by outcome and AR/TE group. ^3^Difference-in-difference estimates for changes in Arkansas and TE states over time. All models incorporated IPTW weights with standard errors clustered at the state level. | | | | | | | | | | | | |

| **Appendix A12. Adjusted changes in health care access for Arkansas and fifteen traditional expansion states, 2011-2021** | | | | | | | | | | | | |
| --- | --- | --- | --- | --- | --- | --- | --- | --- | --- | --- | --- | --- |
| **Outcome** | **Arkansas/ Control^1^** | **Estimated Differences** | | | | | | | | | | |
|  |  | **Initial Waiver vs. Baseline** | |  | **Waiver Extension vs. Baseline** | | |  | **Waiver Extension vs. Initial Waiver** | | | |
|  |  | **Estimate (95% CI)^2^** | **Difference (95% CI)^3^** |  | **Beta^2^** | **Difference^3^** |  |  | **Beta^2^** |  | **Difference^3^** |  |
| Have Insurance Coverage | Arkansas | 20.2*** (17.2, 23.0) | 7.5*** (5.3, 9.7) |  | 26.3*** (23.4, 29.1) | 11.6*** (9.7, 13.6) | |  | 6.1*** (3.1, 9.2) | | 4.2*** (3.3, 5.0) | |
|  | Control | 14.0*** (13.1, 15.0) | -- |  | 16.4*** (15.4, 17.4) | -- | |  | 2.4*** (1.5, 3.2) | | -- | |
| Have Personal Doctor | Arkansas | 5.3** (1.8, 8.8) | 0.1 (-2.2, 2.5) |  | 7.9*** (4.5, 11.3) | 1.2 (-2.1, 4.4) | |  | 2.6 (-0.5, 5.7) | | 1.0 (-0.1, 2.1) | |
|  | Control | 5.9*** (4.9, 7.0) | -- |  | 7.9*** (6.8, 9.0) | -- | |  | 2.0*** (1.1, 2.9) | | -- | |
| Avoided Care Due to Cost | Arkansas | -10.3*** (-13.7, -6.9) | -1.2 (-2.3, 0.0) |  | -14.3*** (-17.5, -11.1) | -0.6 (-1.9, 0.7) | |  | -4.0* (-7.2, -0.8) | | 0.5 (-0.4, 1.5) | |
|  | Control | -7.9*** (-8.8, -7.0) | -- |  | -12.0*** (-12.9, -11.0) | -- | |  | -4.1*** (-4.9, -3.2) | | -- | |
| Last Routine Checkup | Arkansas | 10.1*** (6.5, 13.6) | 5.0*** (3.9, 6.0) |  | 19.3*** (16.0, 22.6) | 10.1*** (7.7, 12.5) | |  | 9.3*** (5.9, 12.6) | | 5.2*** (3.1, 7.2) | |
|  | Control | 5.9*** (4.7, 6.9) | -- |  | 10.5*** (9.4, 11.6) | -- | |  | 4.7*** (3.8, 5.7) | | -- | |
| Flu Vaccine | Arkansas | 4.5* (1.0, 8.0) | 1.3 (-0.4, 3.0) |  | 2.4 (-1.0, 5.8) | -1.6** (-2.7, -0.4) | |  | -2.1 (-5.2, 1.1) | | -2.9*** (-3.9, -1.8) | |
|  | Control | 3.2*** (2.2, 4.2) | -- |  | 4.0*** (3.0, 5.1) | -- | |  | 0.8 (-0.1, 1.7) | | -- | |
| **Source:** Authors' analysis of data from low-income adult respondents to the 2011-2021 Behavioral Risk Factor Surveillance System (BRFSS). **Notes:** The table displays regression-adjusted percentage-point changes in outcomes during each period. Regression estimates are adjusted for respondent age, education, employment status, household size, veteran status, sex, household income, homeownership status, survey month, and whether the survey was conducted via landline or cell phone. The "Baseline" period includes 2011-2013, the "Initial Waiver" period includes 2014-2016, the "Waiver Extension" period includes 2017-2021. ^1^Traditional expansion (TE) states included California, Colorado, Connecticut, Illinois, Kentucky, Minnesota, Nevada, New Jersey, New Mexico, North Dakota, Ohio, Oregon, Rhode Island, Washington, and West Virginia. ^2^Results from logistic regression models stratified by outcome and AR/Control group. ^3^Difference-in-difference estimates for changes in Arkansas and control states over time. All models incorporated BRFSS sampling weights with standard errors clustered at the state level. | | | | | | | | | | | | |

| **Appendix A13. Adjusted changes in health care access for Arkansas and three traditional expansion states, without outcome imputation, 2011-2021** | | | | | | | | | | | | |
| --- | --- | --- | --- | --- | --- | --- | --- | --- | --- | --- | --- | --- |
| **Outcome** | **Arkansas/ Control^1^** | **Estimated Differences** | | | | | | | | | | |
|  |  | **Initial Waiver vs. Baseline** | |  | **Waiver Extension vs. Baseline** | | |  | **Waiver Extension vs. Initial Waiver** | | | |
|  |  | **Estimate (95% CI)^2^** | **Difference (95% CI)^3^** |  | **Beta^2^** | **Difference^3^** |  |  | **Beta^2^** |  | **Difference^3^** |  |
| Have Insurance Coverage | Arkansas | 21.1*** (18.0, 24.2) | 3.4 (0.0, 6.8) |  | 26.6*** (23.5, 29.6) | 4.8** (1.4, 8.2) | |  | 5.5*** (2.3, 8.7) | | 1.5 (-1.8, 4.9) | |
|  | Control | 15.5*** (14.0, 17.0) | -- |  | 19.0*** (17.5, 20.4) | -- | |  | 3.5*** (1.9, 5.1) | | -- | |
| Have Personal Doctor | Arkansas | 7.5*** (3.7, 11.2) | 3.6 (-0.7, 7.8) |  | 9.5*** (6.0, 13.1) | 3.1 (-1.0, 7.1) | |  | 2.1 (-1.2, 5.4) | | -0.5 (-4.2, 3.2) | |
|  | Control | 4.0*** (2.1, 5.8) | -- |  | 6.5*** (4.8, 8.3) | -- | |  | 2.6** (0.9, 4.3) | | -- | |
| Avoided Care Due to Cost | Arkansas | -11.3*** (-14.9, -7.7) | -2.1 (-5.8, 1.7) |  | -14.6*** (-18.1, -11.2) | 0.5 (-3.1, 4.1) | |  | -3.3 (-6.7, 0.1) | | 2.6 (-0.9, 6.1) | |
|  | Control | -8.1*** (-9.7, -6.4) | -- |  | -13.6*** (-15.1, -12.0) | -- | |  | -5.5*** (-7.1, -3.9) | | -- | |
| Last Routine Checkup | Arkansas | 11.7*** (7.8, 15.5) | 6.4** (2.2, 10.6) |  | 20.0*** (16.5, 23.6) | 7.7*** (3.7, 11.8) | |  | 8.4*** (4.8, 11.9) | | 1.3 (-2.5, 5.2) | |
|  | Control | 4.8*** (2.9, 6.7) | -- |  | 11.5*** (9.7, 13.3) | -- | |  | 6.7*** (5.0, 8.4) | | -- | |
| Flu Vaccine | Arkansas | 4.1* (0.4, 7.7) | 2.1 (-2.0, 6.2) |  | 1.9 (-1.7, 5.4) | -0.5 (-4.5, 3.4) | |  | -2.2 (-5.4, 1.0) | | -2.6 (-6.3, 1.0) | |
|  | Control | 2.0* (0.2, 3.8) | -- |  | 2.4** (0.7, 4.1) | -- | |  | 0.4 (-1.2, 2.1) | | -- | |
| **Source:** Authors' analysis of data from low-income adult respondents to the 2011-2021 Behavioral Risk Factor Surveillance System (BRFSS). **Notes:** The table displays regression-adjusted percentage-point changes in outcomes during each period. Regression estimates are adjusted for respondent age, education, employment status, household size, veteran status, sex, household income, homeownership status, survey month, and whether the survey was conducted via landline or cell phone. The "Baseline" period includes 2011-2013, the "Initial Waiver" period includes 2014-2016, the "Waiver Extension" period includes 2017-2021. ^1^Control states included three states that most closely resemble Arkansas in terms of demographics: Kentucky, Ohio, and West Virginia. ^2^Results from logistic regression models stratified by outcome and AR/Control group. ^3^Difference-in-difference estimates for changes in Arkansas and control states over time. All models incorporated BRFSS sampling weights with standard errors clustered at the state level. | | | | | | | | | | | | |
